# *C5* and *SRGAP3* Polymorphisms Are Linked to Paediatric Allergic Asthma in the Italian Population

**DOI:** 10.3390/genes13020214

**Published:** 2022-01-25

**Authors:** Daria Messelodi, Cristina Giuliani, Francesca Cipriani, Silvia Armuzzi, Emanuela di Palmo, Paolo Garagnani, Luca Bertelli, Annalisa Astolfi, Donata Luiselli, Giampaolo Ricci, Andrea Pession

**Affiliations:** 1 Department of Medical and Surgical Sciences (DIMEC), University of Bologna, 40138 Bologna, Italy; daria.messelodi2@unibo.it; 2 Laboratory of Molecular Anthropology, Centre for Genome Biology, Department of Biological, Geological and Environmental Sciences, University of Bologna, 40126 Bologna, Italy; cristina.giuliani2@unibo.it; 3 Pediatric Unit, IRCCS Azienda Ospedaliero-Universitaria di Bologna, 40138 Bologna, Italy; francy.cipriani@gmail.com (F.C.); emanuela.dipalmo@aosp.bo.it (E.d.P.); luca.bertelli@aosp.bo.it (L.B.); andrea.pession@unibo.it (A.P.); 4 Institute of Hematology “Seràgnoli”, IRCCS Azienda Ospedaliero-Universitaria di Bologna, 40138 Bologna, Italy; silvia.armuzzi2@unibo.it; 5 Department of Experimental, Diagnostic and Specialty Medicine (DIMES), University of Bologna, 40138 Bologna, Italy; paolo.garagnani2@unibo.it (P.G.); annalisa.astolfi@unibo.it (A.A.); 6 Department of Translational Medicine, University of Ferrara, 44121 Ferrara, Italy; 7 Laboratory of Ancient DNA (aDNALab), Department of Cultural Heritage (DBC), Ravenna Campus, University of Bologna, 40126 Bologna, Italy; donata.luiselli@unibo.it

**Keywords:** allergy, asthma, allergic rhinoconjunctivitis, single nucleotide polymorphism, allelic distribution

## Abstract

Asthma is a complex and heterogeneous disease, caused by the interaction between genetic and environmental factors with a predominant allergic background in children. The role of specific genes in asthmatic bronchial reactivity is still not clear, probably because of the many common pathways shared with other allergic disorders. This study is focused on 11 SNPs possibly related to asthma that were previously identified in a GWAS study. The genetic variability of these SNPs has been analysed in a population of 773 Italian healthy controls, and the presence of an association between the polymorphisms and the asthma onset was evaluated performing genotyping analysis on 108 children affected with asthma compared with the controls. Moreover, a pool of 171 patients with only allergic rhinoconjunctivitis has been included in the case–control analysis. The comparison of allele frequencies in asthmatic patients versus healthy controls identified two SNPs—rs1162394 (*p* = 0.019) and rs25681 (*p* = 0.044)—associated with the asthmatic condition, which were not differentially distributed in the rhinoconjunctivitis group. The rs25681 SNP, together with three other SNPs, also resulted in not being homogenously distributed in the Italian population. The significantly higher frequency of the rs25681 and rs1162394 SNPs (located, respectively, in the *C5* and *SRGAP3* genes) in the asthmatic population suggests an involvement of these genes in the asthmatic context, playing a role in increasing the inflammatory condition that may influence asthma onset and clinical course.

## 1. Introduction

Asthma is a chronic inflammatory disorder of the airways that represents a relevant health problem, especially for children and young adults. According to the Global Burden of Disease Study, there are almost 300 million people affected by asthma in the world, and at least 10% of all Europeans have to deal with this health issue [1,2,3]. Asthma is a complex and heterogeneous disease, whose causes are not yet clearly identified. Asthma comprises a wide range of subtypes with differences in presentation, pathogenesis and disease course, including allergic and non-allergic asthma, childhood and adult asthma and obesity-related manifestations [4,5]. The major reported risk factors include atopy and atopic dermatitis, microbial exposure, indoor and outdoor allergens, atmospheric pollution and environmental tobacco smoke [6,7,8,9,10]. Because of their heterogeneity and complex interactions, it is difficult to distinguish the role of each component. Among all, genetics plays a key role ranging between 35% and 95% of cases [11], and many studies have already identified hundreds of genes and polymorphic variants supporting this correlation. Most of these genes are regulators of adaptive and innate immune responses or involved in T-cell activation and cutaneous allergic sensitisation [12]. In particular, IL-4, IL-13, IL-17 and IL-33 have been identified as being associated with asthma [13,14,15,16,17]. Other potential involved genes are *HLA*, *CD14* and *ADAM33* playing a key role in different cell functions (differentiation, inflammation and tissue repair) and multiple markers located on chromosome 17q21 regulating the *ORMDL3* expression, which have been reported as being associated with childhood onset asthma [18,19,20,21,22]. To understand the contribution of each genetic risk factor leading to asthma, it is therefore fundamental to stratify the study populations according to the different clinical features. Many are indeed the common genetic architectures shared between asthma and allergy, with high similarities in immune/inflammatory systems and epithelium tissue-related pathways. [23,24].

Based on this observation, in this study we have focused on the genetic components strictly related to asthma in an allergic background, analysing an allergic population stratified into two groups of patients: children with asthma and rhinoconjunctivitis (RC) and children only affected with RC.

A total of 11 single nucleotide polymorphisms (SNPs) were selected among the most significant ones found in a previously published GWAS study, which compared two paediatric populations affected with RC and asthma [25], with the aim of validating their role in allergic asthma onset in a set of independent samples.

Moreover, considering that the Italian peninsula has a peculiar pattern of population structure and a very heterogeneous genomic background, an analysis of the geographic distribution of the 11 SNPs has been performed to detect the presence of a genetic structure among the four different Italian macro-areas (northern, central, southern and Sardinia) and to identify a potential population-specific pattern of genetic variation. Interestingly, in a study conducted on a big cohort of Italian young adults, the occurrence of asthma-like symptoms was reported to be more common in central-southern Italy, rather than in the northern area [26]. Indeed, the description of a possible unbalance in these SNPs’ distribution discloses crucial implications for understanding differential susceptibility to asthma and other inflammatory/autoimmune disorders [27].

## 2. Materials and Methods

### 2.1. Study Design and Populations

The study population includes two main groups of people: an allergic patients’ group and healthy controls.

The allergic group is constituted by 279 children and young adults recruited from 2012 and 2014 with an Italian ancestry coming from multiple Italian regions and a diagnosis of allergic asthma and/or allergic RC. This group was then split in two other subgroups: the asthma population including 108 patients diagnosed with allergic asthma with or without allergic RC and a second subgroup consisting of 171 children with a diagnosis of RC excluding asthma and asthmatic symptoms.

The control group (CTRL) includes 773 Italian people recruited from the Italian population already analysed through the Illumina Bead-Chip 550 k on almost 542,585 genetic markers in the study published by Sazzini et al., 2016 [28].

All the allergic group members were followed as outpatients in the Paediatric Allergologic Unit of S. Orsola-Malpighi Hospital in Bologna. The study was conducted in accordance with the approved guidelines of the Declaration of Helsinki. The study protocol was approved by the Ethical Committee of S. Orsola Malpighi Hospital of Bologna (code 134/2008/U/Tess). Written informed consent was obtained from all study participants or their legal guardians.

The diagnosis of asthma was formulated by a physician on the basis of clinical symptoms, such as wheezing, cough and chest tightness, and spirometric tests. The diagnosis of RC was based on clinical symptoms such as rhinorrhea, sneezing, nasal obstruction and burning in the eyes. A total of 123 children had other pathologies related to allergic diseases such as SOA, bronchial asthma, urticaria, food allergy, latex allergy, allergy to Hymenoptera venom. The diagnosis of allergic disease was performed by skin prick tests (SPTs) with a panel of commercial extracts (ALK-Abello, Italy), including P. pratense (Timothy grass), Cynodon dactylon (Bermuda grass), Chenopodium album (white goosefoot), Betula verrucosa (birch), Cupressus arizonica (cypress), Corylus avellana (hazel), Platanus orientalis (plane tree), Olea europaea (olive tree) and Parietaria judaica (pellitory). Histamine 0.1 mg/mL and glycerol solution were the positive and negative controls, respectively. Morrow Brown needles were used to prick the skin. Readings were taken at 15 min, and wheals ≥3 mm were considered positive. The main clinical characteristics of these populations are reported in Table 1.

### 2.2. SNP Selection

The investigated SNPs were selected from the pooled GWA study of Ricci G. et al. [25]. In total, 11 SNPs were considered among the 24 top scoring SNPs that had shown a greater statistical significance and a preponderant role in the pathophysiology of asthma (Table 2). The selected SNPs have an important role in the airway smooth muscle contraction, bronchoconstriction pathways and in the regulation of inflammation.

### 2.3. DNA Extraction and SNP Genotyping

Peripheral blood was collected from patients at the study enrolment time and DNA was extracted using the QIAamp DNA Mini kit (Qiagen, Hilden, Germany) and quantified through the spectrophotometer NanoDrop (Thermo Fisher Scientific, Waltham, MA, USA).

SNP genotyping analysis was performed in duplicate through the 5′-nuclease TaqMan SNP Genotyping assay (Invitrogen, Waltham, MA, USA) with specific dyes for each selected SNP.

Each DNA sample was diluted to the final concentration of 10 ng/μL and 1 μL was incubated with 4.5 μL of TaqMan Genotyping MasterMix (Applied Biosystems, Waltham, MA, USA), 0.3 μL of the selected dye and 4.2 μL of water. A no template control and 3 samples with a known genotype for each different allelic condition were used as negative and positive controls, respectively. Positive controls were obtained from the pool of samples analysed in Ricci et al. with the Sequenom approach [25].

Fluorescence was read in real-time through the LightCycler480 Real-Time PCR System (Roche, Basel, Switzerland), with the following programme: 95 °C for 10 min, (95 °C for 15 s, 60 °C for 1 min) for 40 cycles, 4 °C for 10 min. Dates were detected in the dual colour hydrolysis probe format and analysed with the Endpoint genotyping module (Figure S1).

### 2.4. Statistical Analyses

Statistical analyses were performed using the software PLINK 1.9 (www.cog-genomics.org/plink/1.9/) [29]. All the SNPs analysed had a genotype with a 100% call rate and satisfy the Hardy–Weinberg equilibrium (HWE) in the control group. To calculate the significance of gender distribution and atopic dermatitis prevalence a Chi squared test was performed. Statistical significance for age at blood sampling and at symptoms onset was evaluated through a Mann–Whitney test. Association analyses (—assoc function) have been performed to compare allele frequencies between cases and controls, and a logistic regression has been calculated with the logistic function. The first comparison was performed between the asthma subgroup vs. CTRL, the second between the RC subgroup vs. CTRL and the third one between the whole allergic population (asthma + RC) vs. CTRL. Odds Ratios (OR) and asymptotic *p*-value were considered for each SNP. *p*-values ≤ 0.05 were considered statistically significant. The allele frequency distribution of the SNPs selected was examined in the four different Italian macro-areas (N_ITA, C_ITA, S_ITA and SARD) according to Sazzini et al. [28].

## 3. Results

### 3.1. Population Description

The study population included 279 paediatric or young adult patients (101 female and 178 male) that were diagnosed with either asthma and/or RC, with or without other allergic manifestations such as atopic dermatitis or food allergy. The patients were stratified into two groups: 108 showing asthma with or without RC and 171 affected with RC without asthma. Clinical and phenotypic data of the subjects recruited in the allergic group are summarised in Table 1. Among the patients of the two groups no differences apart from the male gender were associated with the asthmatic phenotype (*p* = 0.02). The mean age at first diagnosis for the RC group was 8.2 ± 4.0 years, while in the asthmatic group it was 8.7 ± 3.8 years, a difference that is in line with the “atopic march” theory [30] but is not statistically significant. The age of patients at study recruitment depends on the time of patient clinical follow-up and is not significantly different between the RC (12.0 ± 3.8 years) and asthma (13.0 ± 3.6 years) groups.

### 3.2. C5 and SRGAP3 SNPs Are Risk Factors for Asthma Manifestation

Genotyping data was collected from all the patients of the asthma and RC group for the 11 selected SNPs, and an association analysis was performed in order to find a correlation between the allele frequency and the presence of asthma/allergic conditions in the population.

To identify the presence of associations strictly related to the asthmatic population, the variability in the allele frequencies between asthma (*n* = 108) and CTRL (773) groups was evaluated.

Two SNPs were found associated to asthma with a significant *p*-value: rs25681 in *C5* gene (*p* = 0.044, OR = 1.34) and rs1162394 in *SRGAP3* (*p* = 0.019, OR = 1.41) (Table 3). Considering that sex was found to be associated with asthma in the whole allergic population, a logistic regression was performed adding sex as a covariate, and only rs1162394 in the *SRGAP3* gene shows a significant nominal *p*-value (*p* = 0.015).

In order to evaluate if the same polymorphisms were also related to the onset of an RC condition, the association analysis was performed comparing 171 patients of the RC group with the CTRL and no significant differences were observed (Table S1), suggesting that the previously identified associations were strictly characteristics of the asthmatic component of the allergic population and not related to the allergic rhinitis subgroup. As a further confirmation, the comparison carried out on the whole allergic population (*n* = 279) vs. CTRL showed an association just with rs1162394 in the *SRGAP* gene with a reduced significance (*p* = 0.032, OR = 1.24), implying that the association driven by the asthmatic component of the population is partially lost considering the whole allergic population (Table S2).

### 3.3. Allelic Distribution in Italy Is Not Homogeneous for Four SNPs, Including rs25681 in C5 Gene

To evaluate if the distribution of the analysed SNPs is homogenous in Italy, their allelic frequencies was analysed in the CTRL population in the four different macro-areas: north, central, south and Sardinia. Italy has a well proven heterogeneous genomic background regarding immune pathways, which due to adaptive events to peculiar pathogen landscapes, caused an increased susceptibility to autoimmune or inflammatory disorders [28].

The comparisons carried out among the different CTRL subsamples highlighted significant nominal *p*-values for four SNPs: rs4580655 (*TACR3*), rs10760153 (*RAB14*), rs12820238 (*EPYC*) and, interestingly, also rs25681 (*C5*) (Figure S2). *TACR3*, *RAB14* and *C5* SNPs showed genetic differentiation in the comparisons between individuals from the Italian peninsula with the Sardinia group which is known to be the most genetically distant group in Italy [31], while rs12820238 was differentially distributed in the comparison between northern and southern Italy (Table 4).

## 4. Discussion

One important feature of asthma is the strict connection with other allergic diseases such as rhinitis, food allergies and atopic dermatitis. There is a wide interest of the scientific community in identifying the genetic factors that foster the onset of the disease, and in understanding the relationship among the different risk factors. Asthma and RC are often considered as a diverse expression of an allergic phenotype, even if not everyone who shows RC will eventually develop asthma during life. To search for possible genetic determinants, we have divided patients with only allergic RC from patients with allergic asthma (with or without RC) in order to reduce the impact of the allergic genetic background. From a previous study carried out with the same experimental design [25], we selected 11 SNPs to try to validate their connection with asthma in a set of independent samples. According to statistical analysis, 2 out of the 11 analysed SNPs were associated to asthma with a significant *p*-value: rs1162394 (gene *SRGAP3*) and rs25681 (gene *C5*), while both SNPs resulted in being not linked to RC manifestation. It is important to consider that the healthy CTRL group is made of Italian blood donors, and it could include people suffering from light forms of allergic rhinitis since it is not generally considered a real pathologic condition. This might cause an underestimation of the case frequencies in the comparison between RC and CTRL groups but it does not influence the analysis involving the asthma group. We are also aware that the reported *p*-values are nominal, and the low sample size of this study could increase the possibility of a false positive; therefore, future studies are needed to confirm these associations. rs25681 is located on *C5*, a gene with a consolidated role in asthma and allergic sensitisation [32,33,34,35] It is known that complement effectors are involved in the onset of many pathophysiological features of asthma, including inflammatory cell infiltration, increases in vascular permeability and smooth muscle cell contraction [36]. Experiments carried out in murine asthma models have shown a reduction in the inflammatory response when the complement factor *C5* inhibition occurred [37,38,39], opening to a therapeutic approach aiming to reduce complement-mediated airway injury.

*SRGAP3* was never related to asthma or RC until the GWAS study carried out in 2011 [25], and up to now no studies have reported its role in asthma or any allergic diseases. *SRGAP3* belongs to the family of Rho GTPase protein and is a regulator of the cytoskeleton dynamics. Interestingly, *SRGAP3* works by downregulating the Rho GTPase Rac1 signalling [39]. Rac1 has been recently identified as an important player in the regulation of intracellular calcium and airway smooth muscle cell contraction, and its role as a therapeutic target in asthma is under evaluation [40].

The presence of a genetic structure for the above-mentioned SNPs in the Italian population was also investigated in this study. This approach is particularly interesting considering the heterogeneity of the genetic background in Italy. Even if we do not have a genome-wide analysis that would have allowed to reconstruct the genetic structure of the cases, it is conceivable that they are uniform to controls and come from all over Italy. Moreover, studies on the evolution of the human immune system and autoimmune disease in primates highlighted the presence of signatures of the positive selection of immune genes that correspond to asthma susceptibility loci in humans [41], suggesting that airway inflammatory disorder predisposition can have an unbalanced geographic distribution.

The 11 SNPs were analysed to detect allelic frequency distribution in four Italian macro-areas [28], highlighting four differentially distributed SNPs: rs4580655 (*TACR3*), rs25681 (*C5*), rs10760153 (*RAB14*) and rs12820283 (*EPYC*). These SNPs are located in genes whose functions are strongly related to inflammation activation mechanisms: *C5*, as already discussed, is a pro-inflammatory mediator in asthma conditions; the *RAB14* gene regulates the endocytic transport of ADAM10, which influences the generation of Th2 immune responses [42]; *TACR3* encodes for neurokinin B, involved in asthma pathophysiology by mediating neurogenic inflammation and lung functionality [43]. Three of them showed a significantly higher allele frequency among Sardinians, who are known to have a distinct genetic signature if compared with the whole European genetic landscape [44,45]. The unbalanced allelic distribution probably does not have a consistent impact on the development of allergic disease symptomatology since in just one case the risk allele is more represented in this population. The other SNP, rs12820238, resulted differentially distributed in southern Italy when compared with northern Italy, with a higher frequency of the risk allele in the south compared to the other areas. In a previous study focusing on the role of climate on the geographic variability of asthma and respiratory symptoms in Italy, an increase of asthma-like symptoms in central-southern Italy was already reported. This was widely explained considering the effect of many environmental factors but without considering a possible genetic impact [26]. Moreover, this trend is in line with the previously identified genetic signature of local adaptation in genes involved in inflammatory and autoimmune diseases in southern Italy [28,45].

In summary, this validation analysis confirmed the implication of rs25681 in the *C5* gene in the inflammatory context of asthma. In addition, the *SRGAP3* gene was identified as a candidate susceptibility gene. The genetic variability of these polymorphisms seems to be associated with an increased risk of asthma but not RC in children. Despite the fact that the obtained results require confirmatory analyses to validate the highlighted associations, this study represents the first exploratory investigation of the distribution of genetic risk factors for asthma in Italy. This validation study of previously collected GWAS data confirms once again the presence of peculiar genetic traits of asthmatic patients, whose identification could, in the future, pave the way for the development of more targeted clinical interventions.

## Figures and Tables

**Table 1:** Main clinical characteristics of the allergic children group included in the study (*n* = 279).

	Asthma (*n* = 108)	RC (*n* = 171)	*p*-Value
Sex (male/female)	2.6	1.4	0.02
Diagnosis age (mean years ± SD)	8.7 ± 3.8	8.2 ± 4.0	0.55
Recruitment age (mean years ± SD)	13.0 ± 3.6	12.0 ± 3.8	0.51
Atopic dermatitis (%)	34.2	38.0	0.52
Other allergic pathologies (%)	41.6	45.6	0.63

**Table 2:** Candidate SNPs investigated in this study, selected among the 24 SNPs identified as associated with asthma through the GWAS study of Ricci et al. [22] (dbSNP: variant identifier; Chr: chromosome localisation; BP: Genomic coordinate).

dbSNP	Chr	BP	Minor Allele	Gene Symbol	Description	Risk Allele	Risk Genotype
rs10754593	1q43	237326348	G	*RYR2*	Ryanodine receptor 2	G	C/G + G/G
rs1162394	3p25.3	9125744	C	*SRGAP3*	SLIT-ROBO Rho GTPase activating-protein 3	C	C/C + C/G
rs694936	3q12.3	101587793	T	*NFKBIZ*	Nuclear factor of kappa light polypeptide gene enhancer in B-cells inhibitor, zeta	A	A/A
rs1456114	3q21.1	123245152	G	*PTPLB*	Protein tyrosine phosphatase-like (proline instead of catalytic arginine), member b	G	A/G + G/G
rs4580655	4q24	104529274	G	*TACR3*	Tachykinin receptor 3	G	G/G
rs456290	5q33.3	156155155	C	*SGCD*	Sarcoglycan, delta (35 kDa dystrophin-associated glycoprotein)	C	C/C + C/T
rs3250	7q33	134845432	C	*TMEM14, C7orf49*	*TMEM140*/chromosome 7 open reading frame 49	C	C/C + T/T
rs531003	9q31.3	113612619	C	*LPAR1*	Lysophosphatidic acid receptor 1	G	G/G
rs25681	9q3302	123780005	T	*C5*	Complement component 5	C	C/C
rs10760153	9q33.2	123948375	C	*RAB14*	RAS oncogene family member	T	T/T
rs12820238	12q21.33	91356040	T	*C12orf12, EPYC*	Chromosome 12 open reading frame 12/epiphycan	T	T/T + G/T

**Table 3:** Comparison of allele frequencies of the 11 SNPs in the asthma (*n* = 108—cases) and CTRL (*n* = 773) groups. (A1: minor allele; F_A: frequency among cases; F_U: frequency among controls; A2: major allele; CHISQ: allelic test chi-square statistic; *p*: allelic *p*-value; OR: odds (allele 1|cases)/odds (allele 1|controls)). Significant associations (nominal *p*-values) are highlighted in bold.

dbSNP	Gene	A1	F_A	F_U	A2	CHISQ	*p*	OR
rs10754593	*RYR2*	“G”	0.412	0.395	“C”	0.24	0.62	1.08
**rs1162394**	***SRGAP3***	**“C”**	**0.482**	**0.398**	**“G”**	**5.49**	**0.019**	**1.41**
rs694936	*NFKBIZ*	“T”	0.440	0.436	“A”	0.01	0.91	1.02
rs1456114	*PTPLB*	“G”	0.324	0.345	“A”	0.36	0.55	0.91
rs4580655	*TACR3*	“G”	0.394	0.391	“A”	0.0064	0.94	1.01
rs456290	*SGCD*	“C”	0.144	0.157	“T”	0.29	0.59	0.89
rs3250	*TMEM14*	“C”	0.468	0.450	“T”	0.23	0.63	1.07
rs531003	*LPAR1*	“C”	0.282	0.263	“G”	0.38	0.54	1.11
**rs25681**	***C5***	**“T”**	**0.509**	**0.437**	**“C”**	**4.05**	**0.044**	**1.34**
rs10760153	*RAB14*	“C”	0.500	0.452	“T”	1.80	0.18	1.22
rs12820238	*EPYC*	“T”	0.185	0.148	“G”	2.09	0.15	1.31

**Table 4:** Features of SNPs with a statistically significant genetic variability among the four Italian macro-areas (A1 = minor allele, N_ITA = northern Italy, C_ITA = central Italy, S_ITA = southern Italy, SARD = Sardinia).

dbSNP	A1	Comparisons	Frequencies Group (1)	Frequencies Group (2)	CHISQ	*p*-Value (Nominal)	OR (CI 95%)
rs4580655	G	N_ITA (1) vs. SARD (2)	0.339	0.500	7.93	0.0048	1.95 (1.22–3.10)
		C_ITA (1) vs. SARD (2)	0.393	0.500	4.18	0.041	1.54 (1.02–2.33)
rs25681	T	N_ITA (1) vs. SARD (2)	0.455	0.580	4.54	0.033	1.65 (1.04–2.63)
		C_ITA (1) vs. SARD (2)	0.418	0.580	9.51	0.002	1.92 (1.26–2.92)
		S_ITA (1) vs. SARD (2)	0.425	0.580	7.37	0.0066	1.87 (1.19–2.94)
rs10760153	C	S_ITA (1) vs. SARD (2)	0.459	0.580	4.44	0.035	1.63 (1.03–2.56)
rs12820238	T	N_ITA (1) vs. S_ITA (2)	0.115	0.178	4.47	0.034	1.66 (1.03–2.65)

## Data Availability

Data is contained within the article or Supplementary Material. Additional data presented in this study are available on request from the corresponding author.

## Supplementary Materials

The following are available online at https://www.mdpi.com/article/10.3390/genes13020214/s1, Figure S1: Allelic discrimination plots resulting from the SNP genotyping assay for the 11 analysed SNPs, Figure S2: Representation of the allelic distribution for the rs25681 SNP in *C5* gene in the four Italian macro-areas, Table S1: Comparison of SNP allele frequencies in RC group (*n* = 171—cases) and CTRL (*n* = 773), Table S2: Comparison of SNP allele frequencies in allergic group (*n* = 279—cases) and CTRL (*n* = 773).
