# Supplementary material for: C5 and SRGAP3 Polymorphisms Are Linked to Paediatric Allergic Asthma in the Italian Population
Source: Genes (Basel). 2022 Jan 25;13(2):214. doi: 10.3390/genes13020214 (PMC8871526; doi:10.3390/genes13020214)

Supplementary Figures and Tables

Table S1. Comparison of SNPs allele frequencies in RC group (n=171 - cases) and CTRL (n=773). *(dbSNP: variant identifier; A1: effect allele; F_A: frequency among cases; F_U: frequency among controls; A2: non-effect allele; CHISQ: allelic test chi-square statistic; P: allelic p-value; OR: odds (allele 1| cases)/odds (allele 1| controls))*. No significant association (nominal p-values) was detected.

dbSNP	Gene	A1	F_A	F_U	A2	CHISQ	P	OR
**rs10754593**	RYR2	"G"	0.401	0.395	"C"	0.04	0.84	1.03
**rs1162394**	SRGAP3	"C"	0.430	0.398	"G"	1.19	0.20	1.14
**rs694936**	NFKBIZ	"T"	0.456	0.436	"A"	0.46	0.50	1.09
**rs1456114**	PTPLB	"G"	0.322	0.345	"A"	0.67	0.41	0.90
**rs4580655**	TACR3	"G"	0.383	0.391	"A"	0.069	0.79	0.97
**rs456290**	SGCD	"C"	0.140	0.158	"T"	0.65	0.42	0.87
**rs3250**	TMEM14	"C"	0.439	0.450	"T"	0.15	0.70	0.95
**rs531003**	LPAR1	"C"	0.269	0.263	"G"	0.06	0.81	1.03
**rs25681**	C5	"T"	0.447	0.437	"C"	0.13	0.72	1.05
**rs10760153**	RAB14	"C"	0.439	0.452	"T"	0.19	0.66	0.95
**rs12820238**	EPYC	"T"	0.155	0.148	"G"	0.12	0.72	1.06

Table S2: Comparison of SNPs allele frequencies in allergic group (n=279 - cases) and CTRL (n=773);*A1: effect allele; F_A: frequency among cases; F_U: frequency among controls; A2: non-effect allele; CHISQ: allelic test chi-square statistic; P: allelic p-value; OR: odds (allele 1| cases)/odds (allele 1| controls)).* Significant associations (nominal p-values) are highlighted in bold.

dbSNP	Gene	A1	F_A	F_U	A2	CHISQ	P	OR
**rs10754593**	RYR2	"G"	0.403	0.395	"C"	0.13	0.72	1.04
**rs1162394**	**SRGAP3**	**"C"**	**0.450**	**0.398**	**"G"**	**4.58**	**0.032**	**1.24**
**rs694936**	NFKBIZ	"T"	0.450	0.436	"A"	0.32	0.57	1.06
**rs1456114**	PTPLB	"G"	0.321	0.345	"A"	1.05	0.31	0.90
**rs4580655**	TACR3	"G"	0.389	0.391	"A"	0.0056	0.94	0.99
**rs456290**	SGCD	"C"	0.142	0.157	"T"	0.83	0.36	0.88
**rs3250**	TMEM14	"C"	0.446	0.450	"T"	0.026	0.87	0.98
**rs531003**	LPAR1	"C"	0.274	0.263	"G"	0.28	0.60	1.06
**rs25681**	C5	"T"	0.471	0.437	"C"	2.00	0.16	1.15
**rs10760153**	RAB14	"C"	0.462	0.452	"T"	0.20	0.66	1.05
**rs12820238**	EPYC	"T"	0.167	0.148	"G"	1.17	0.28	1.16

Figure S1: Allelic discrimination plots resulting from the SNP genotyping assay for the 11 analysed SNPs.

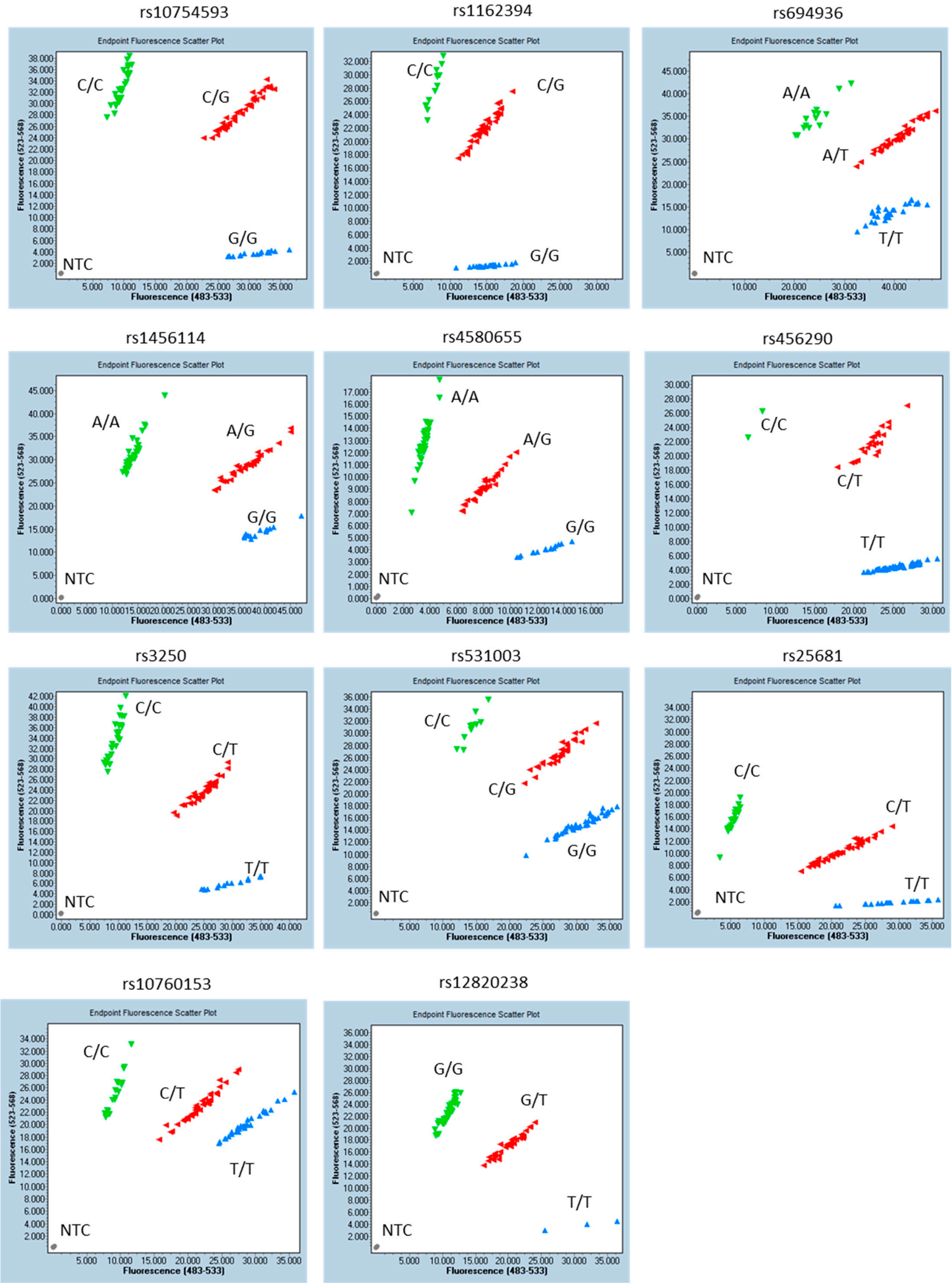

Figure S2. Representation of the allelic distribution for the rs25681 SNP in *C5* gene in the four Italian macro-areas (*N_ITA=northern Italy, C_ITA=central Italy, S_ITA=southern Italy, SARD=Sardinia).*

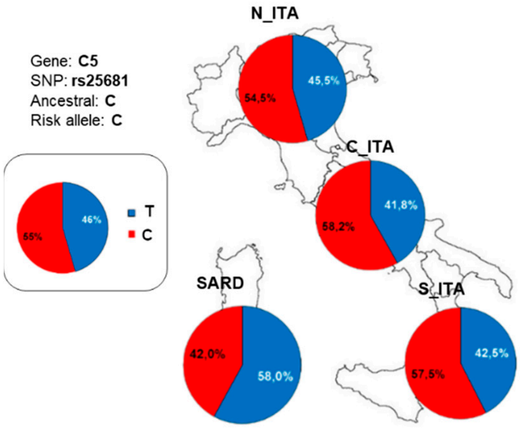

Supplement: Supplementary file 1 [file genes-13-00214-s001.zip › genes-1530318-supplementary.pdf]
